# Bond Graph Modeling and Validation of an Energy Regenerative System for Emulsion Pump Tests

**DOI:** 10.1155/2014/289839

**Published:** 2014-05-22

**Authors:** Yilei Li, Zhencai Zhu, Guoan Chen

**Affiliations:** School of Mechanical and Electrical Engineering, China University of Mining and Technology, Xuzhou 221116, China

## Abstract

The test system for emulsion pump is facing serious challenges due to its huge energy consumption and waste nowadays. To settle this energy issue, a novel energy regenerative system (ERS) for emulsion pump tests is briefly introduced at first. Modeling such an ERS of multienergy domains needs a unified and systematic approach. Bond graph modeling is well suited for this task. The bond graph model of this ERS is developed by first considering the separate components before assembling them together and so is the state-space equation. Both numerical simulation and experiments are carried out to validate the bond graph model of this ERS. Moreover the simulation and experiments results show that this ERS not only satisfies the test requirements, but also could save at least 25% of energy consumption as compared to the original test system, demonstrating that it is a promising method of energy regeneration for emulsion pump tests.

## 1. Introduction

The emulsion pump, one of hydraulic applications, has been widely applied in industry because it has the advantages of common hydraulic oil pump. As a result, the emulsion pump has gradually become the necessary equipment in many industries.

In coal mine industry, for instance, the emulsion pump is necessarily employed on the fully mechanized coal mining face as a hydraulic source of the hydraulic support which is used to support the working space underground and therefore to keep mine workers' lives safe. Consequently, the emulsion pump should be strictly tested before its field usages according to the coal industry standard MT/T 188.2–2000 of China. The original test system used in the test standard is simple but energy-demanding, which uses an induction motor as the direct driver of test pump and employs a throttle valve to set the required load pressure of the test emulsion pump, wasting all the energy during the test. There are several kinds of tests in the standard, such as zero-load test, half-load test, and full-load test. Among all these tests, the durable test costs and wastes the most energy because it requires that the pump be tested continuously under full-load pressure for 500 hours, take, for example, the most commonly used emulsion pump in coal mine of which the nominal pressure and flow rate are 31.5 MPa and 400 L/min. One durable test of such an emulsion pump would cost 105 000 kW·Ch. Moreover, the heat energy converted by the throttle valve and then contained in the emulsion fluid may need a particular radiating system to be dissipated, costing more energy again. As a result, from the environmental point of view, especially energy saving, the original test system is facing serious challenges due to its large energy consumption and low energy efficiency. The ERS has great potential to solve this problem.

Researches on ERS to date have generally been diverse, focusing on variable systems of hydraulic oil, such as hydraulic elevators [1, 2] and hydraulic excavators [3–6], as well as hydraulic forklifts and cranes [7, 8]. These ERSs for hydraulic oil system, however, are not suitable to the emulsion pump tests because of the working fluid difference, not hydraulic oil but emulsion, resulting in no industry productions available in market, like emulsion motor. ERSs [4, 9–11] employing accumulators as the energy-saving unit are not available options because they are not fit for continuous operation systems without working condition changes.

Aiming at the decrease of energy consumption, a novel ERS for emulsion pump tests has been proposed in [12]. Bond graph would be a preferable method for investigating this ERS, since it contains some energy conversions among mechanical translational, rotational, and hydraulic domains.

Bond graph modeling, a graphical methodology particularly suited for modeling multidisciplinary dynamic engineering systems in which components from different disciplines dynamically interact by exchanging energy and in which different forms of energy are involved, was first conceptualized by Paynter [13] in 1959 and then elaborated by Karnopp et al. [14] into a unified modeling methodology that is popular in variable research areas of many physical domains [15–19]. Apart from being a graphical, port-based modeling approach, one advantage of bond graph for ERSs lies in the fact that it is domain-independent, using unified notations for elements and variables across various energy domains; hence, it is capable of representing a complex system involving diverse energy domains. Bond graph has been applied in modeling various complex mechanical systems, such as railway trucks [20], automobiles [21, 22], and aircrafts [23]. Bond graph methodology has also been used for modeling and investigating different kinds of hydraulic systems [24–28]. Borutzky et al. [29] developed bond graph models of some hydraulic components in Modelica software. Renewable energy systems, such as wave and wind energy conversion system, have been modeled based on bond graph in [30, 31].

The main objectives of this paper are the modeling of this ERS for emulsion pump tests using bond graph approach and validation. The remainder of this paper is organized as follows. In the second section, the overall structure of the ERS is briefly described at first. Then bond graph model of this ERS is established by first considering the separate components before assembling them together, and the state-space equations are derived accordingly. In the third section, simulations of the bond graph model are performed within MATLAB/Simulink software. The ERS model and simulation are then validated in the fourth section using experimental data. Conclusions are summarized in the final section.

## 2. Bond Graph Modeling

### 2.1. ERS Description

**Figure 1:**
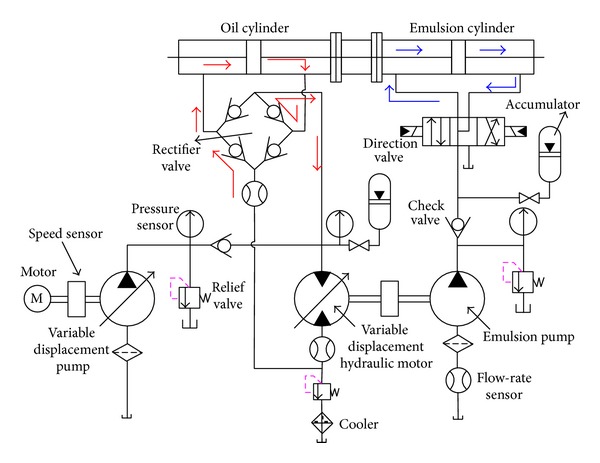
Schematic diagram of the novel energy regenerative system.

The novel ERS for emulsion pump tests, as shown in Figure 1, consists of induction motor, variable displacement pump (VDP), variable displacement hydraulic motor (VDHM), direction control valve, a couple of symmetrical cylinders, rectifier valve, and several kinds of sensors.

The induction motor and its connected VDP serve as the energy source of the whole system, converting electric energy into hydraulic energy. The VDHM which is actuated by the output oil of both VDP and the rectifier valve drives the test emulsion pump, converting the hydraulic energy of oil first into mechanical rotational energy and then back into hydraulic energy of emulsion fluid. The output emulsion fluid from emulsion pump, through the direction valve, flows into the left chamber of emulsion cylinder and pulls the piston of oil cylinder rightward via the common piston rod. Therefore, the oil in the right chamber of oil cylinder is extruded out through the rectifier valve and back into VDHM. By the two cylinders, the output hydraulic energy of emulsion fluid is converted first into mechanical energy of piston and then back into hydraulic energy of oil, implementing the energy regeneration function of this system. As crucial components of this system, the two cylinders not only convert and save energy from emulsion fluid to hydraulic oil, but also simultaneously isolate these two kinds of fluid strictly from each other to avoid mutual contamination. From the energy conversion point of view, the left oil cylinder functions more like a pump with linear motion. The fully detailed working principle of this ERS could be referred to as in the previous paper [32].

### 2.2. Components Modeling

Since the system consists of many hydraulic components and energy conversion processes, it would be much easier to model each single component of the system at first and then assemble them together accordingly. Therefore the decomposition of the whole system into subsystem models exchanging energy is performed before the modeling of the whole system.

#### 2.2.1. Variable Displacement Pump

**Figure 2:**
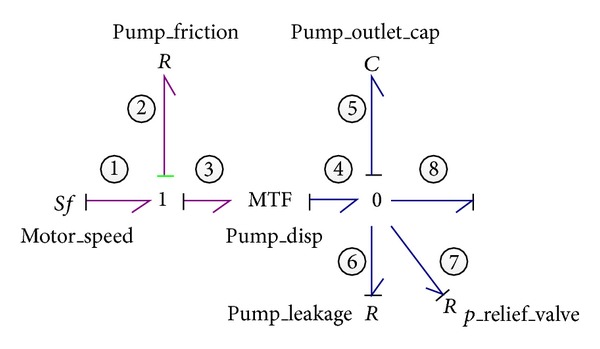
Bond graph model of the variable displacement pump.

The VDP converts the energy from mechanical rotational domain into hydraulic domain and generally serves as a hydraulic power source to the whole system. A detailed model of VDP is established in order to obtain a better dynamic behavior of the pump in which the rotational friction and internal leakage of the pump as well as the fluid compressibility inside the pump are all well considered, as depicted in Figure 2. To simply and clearly denote the power bonds and elements, all the bonds are numbered consecutively and the element attached to the bond is numbered accordingly. Also as preference, both the effort (*e*) and flow (*f*) pointing into the 0-junction and 1-junction are signed as positive while those pointing away from the 0-junction and 1-junction as negative.

The induction motor is simply represented by a velocity source element of *Sf*_1_, assuming that it rotates continuously and steadily at a constant angular speed. In addition, due to the steady input angular speed of pump, the inertia moment effect of the pump is reasonably neglected. The transform ratio of MTF, which is the pump displacement, can be modulated by an input control signal, and the constitutive relation of MTF could be expressed as follows:

(1) $$\begin{array}{c} f_{4}=f_{3}\times V_{p}\left(\alpha \right),\\ e_{3}=e_{4}\times V_{p}\left(\alpha \right), \end{array}$$

where *e*_4_ and *f*_4_ are the pressure and flow rate of the pump, respectively; *e*_3_ and *f*_3_ are the torque and angular velocity of induction motor, respectively; *V*_*p*_(*α*) is the geometric displacement of the pump; and *α* is the control signal of pump displacement.

Clearly, in a realistic model, energy losses inside the component have to be accounted for. In a hydraulic system or component, these losses are mainly pressure drops, leakages, and frictions and can be represented by the resistive element *R* in bond graph modeling. The rotational friction loss and leakage loss, in other words, the mechanical and volumetric coefficients, are implemented with simple resistive elements *R*_2_ and *R*_6_, respectively, while the capacitance effect which occurs both inside the pump and at the outlet due to fluid compressibility is symbolized by an energy store element of *C*_5_. The compressed fluid volume represented by the element *C*_5_ consists of both the pump displacement and the internal space of the pipe connected to the pump outlet.

Additionally, the relief valve employed at the pump outlet for safety is generally represented by a resistive element *R*_7_ with nonlinear resistance. In most cases the internal dynamics of the relief valve could be neglected because its response speed is much faster than any other components of the hydraulic system. The constitutive relation of the *R*_7_ is described as

(2) $$\begin{array}{c} f_{7}=\{\begin{array}{cc} 0 & \text{if}\;\;e_{7}\leq p_{\text{set}}\\ \frac{\left(e_{7}-p_{\text{set}}\right)}{R_{7}} & \text{if}\;\;e_{7}>p_{\text{set}}, \end{array} \end{array}$$

where *f*_7_ and *e*_7_ are the flow rate and pressure of the relief valve, respectively, and *R*_7_ and *p*_set_ are the resistance and predefined pressure of the relief valve, respectively.

In bond graph methodology, the time integral of effort of the *I* element (known as generalized momentum *P*) and the time integral of flow of *C* element (known as generalized displacement *Q*) are usually chosen for system state variables. After the completion of causality assignment, the state-space equation of the VDP is derived as follows:

(3) $$\begin{array}{c} {\dot{Q}}_{5}=-\frac{1}{C_{5}}\left(\frac{1}{R_{6}}+\frac{1}{R_{7}}\right)Q_{5}+V_{p}f_{1}-f_{8}, \end{array}$$

where *Q*_5_ is the oil volume;$\;{\dot{Q}}_{5}$ is the flow rate (the time differential of *Q*_5_); and *f*_1_ and *f*_8_ are the induction motor velocity and pump output flow rate, respectively.

#### 2.2.2. Variable Displacement Hydraulic Motor

**Figure 3:**
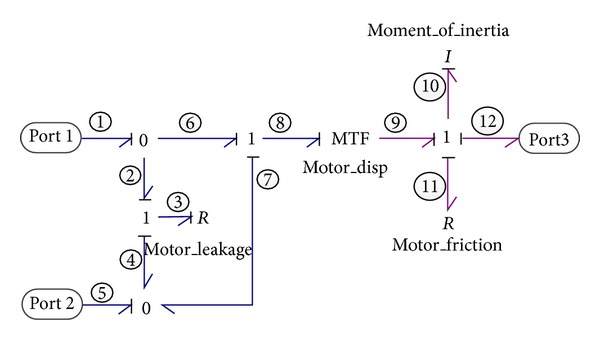
Bond graph model of the variable displacement motor.

In this system, the VDHM, functioned as the actuator of the emulsion pump, converts the hydraulic energy of oil into mechanical rotational domain. As shown in Figure 3, the bond graph model of VDHM consists of three power interchange ports with the environment: the first two ports are the hydraulic inflow and outflow ports of motor, while the third port represents the mechanical connection to the next components, the emulsion pump. Like the VDP model, the internal leakage and rotational friction effects are taken into consideration. Besides, the inertia moment of the motor is also modeled due to the possible fluctuations of rotation velocity and is represented by an energy store element of *I*_10_. The constitutive relations of MTF element symbolizing the variable displacement motor can be expressed as

(4) $$\begin{array}{c} e_{9}=e_{8}\times V_{m}\left(\beta \right),\\ f_{8}=f_{9}\times V_{m}\left(\beta \right), \end{array}$$

where *e*_8_ and *f*_8_ are the input pressure and flow rate of VDHM, respectively; *e*_9_ and *f*_9_ are the output torque and angular velocity of VDHM, respectively; and *V*_*m*_(*β*) and *β* are the motor geometric displacement and displacement control signal, respectively.

The state-space equation of VDHM derived from its bond graph model can be expressed as

(5) $$\begin{array}{c} {\dot{P}}_{10}=-\frac{R_{11}}{I_{10}}P_{10}+V_{m}e_{1}-V_{m}e_{5}-e_{12}, \end{array}$$

where *P*_10_ is the angular momentum; ${\dot{P}}_{10}$ is the torque (the time differential of *P*_10_); and *e*_12_ is the output torque of hydraulic motor.

#### 2.2.3. Emulsion Pump

**Figure 4:**
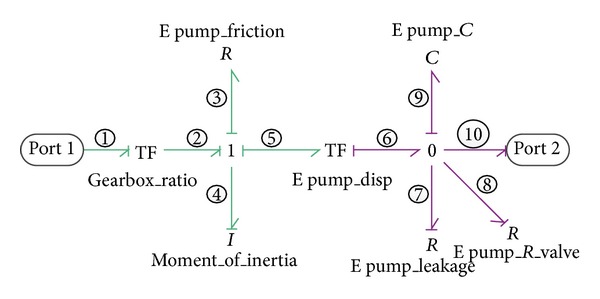
Bond graph model of the emulsion pump.

By the emulsion pump, the input mechanical rotational energy is converted into hydraulic energy of emulsion fluid. As illustrated in Figure 4, the model of emulsion pump is similar to that of VDP except that the emulsion pump is a fixed displacement pump represented by a TF element instead of an MTF and that the emulsion pump contains an internal gearbox. The gearbox can be modeled by another TF element of which the constitutive equation is expressed as

(6) f2=f1×i,e1=e2×i,

where *f*_1_ and *f*_2_ are the input and output angular velocity of the gearbox, respectively; *e*_1_ and *e*_2_ are the input and output torque of the gearbox, respectively; and *i* is the transmission ratio of the gearbox.

Similar to the VDHM model, the inertia moment of emulsion pump is added to the model for the consideration that the rotation velocity may fluctuate during operation.

After causality assignment, the state-space equations of the emulsion pump are obtained as follows:

(7) $$\begin{array}{c} {\dot{P}}_{4}=-\frac{R_{3}}{I_{4}}P_{4}-\frac{V_{\text{ep}}}{C_{9}}Q_{9}+ie_{1},\\ {\dot{Q}}_{9}=\frac{V_{\text{ep}}}{I_{4}}P_{4}-\left(\frac{1}{R_{7}}+\frac{1}{R_{8}}\right)\frac{1}{C_{9}}Q_{9}-f_{10}, \end{array}$$

where *P*_4_ and ${\dot{P}}_{4}$ are the angular momentum and torque, respectively; *Q*_9_ and ${\dot{Q}}_{9}$ are the fluid volume and flow rate, respectively; and *e*_1_ and *e*_10_ are the input torque and output pressure of emulsion pump, respectively.

#### 2.2.4. Direction Control Valve

**Figure 5:**
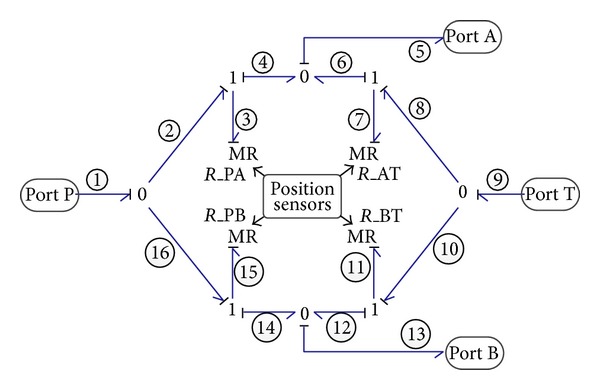
Bond graph model of the direction valve.

To cycle the operation of this system, the 4/3 way direction control valve is used to reverse the flow direction of cylinder chambers when the cylinder piston reaches its end of stroke. The valve contains four ports: ports P and T are linked to pressurized oil and the tank, respectively, while ports A and B are connected to the two chambers of cylinder. The valve has only two working positions (left and right positions) and is controlled to change position by the position sensors installed on the cylinder rod. When the valve works at its left position, the flow paths inside the valve from port P to port A and from port B to port T are open, whereas the flow paths from port P to port B and from port A to port T are sealed. When the valve is triggered to change to its right working position, the flow paths pattern changes reversely. This kind of working principle can be implemented employing four position-modulated dissipative elements of MR between the ports of PA, AT, PB, and BT, the resistance of which is modulated by the control signal from the position sensors, as shown in Figure 5. When the MR resistance between ports P and A, for example, is set to a sufficiently large value, the flow path from ports P to A is closed and hence no fluid passes. A relatively much smaller resistance of MR between port P and port A means that the flow path from port P to port A is open. The constitutive equation of MR can be expressed as follows:

(8) $$\begin{array}{c} f=\{\begin{array}{cc} C_{d}A\sqrt{\frac{2\Delta p}{\rho }} & \text{if}\;\text{flow}\;\text{path}\;\text{is}\;\text{open}\\ 0 & \text{if}\;\text{flow}\;\text{path}\;\text{is}\;\text{closed}, \end{array} \end{array}$$

where *f* is the flow rate; *C*_*d*_ is the discharge coefficient of valve; *A* is the flow area; Δ*p* is the pressure difference between two ports; and *ρ* is the fluid density.

According to the bond graph methodology, the output flow rate of the four ports can be derived and expressed as

(9) f1=(1MR3+1MR15)e1−1MR3e5−1MR15e13,f5=1MR3e1−(1MR3+1MR7)e5+1MR7e9,f9=−1MR7e5+(1MR7+1MR11)e9−1MR11e13,f13=1MR15e1+1MR11e9−(1MR15+1MR11)e13,

where *f*_1_, *f*_5_, *f*_9_, and *f*_13_ are the flow rate of ports P, A, T, and B, respectively and *e*_1_, *e*_5_, *e*_9_, and *e*_13_ are the pressure of ports P, A, T, and B, respectively.

#### 2.2.5. Symmetrical Cylinders

A hydraulic cylinder is essentially a power transformer which converts the hydraulic power into mechanical translational domain and could be simply symbolized by a TF element in bond graph modeling with the following constitutive equations:

(10) $$\begin{array}{c} F=p\times A,\\ q=v\times A, \end{array}$$

where *F* and *v* are the output force and velocity of cylinder, respectively; *p* and *q* are the input pressure and flow rate of cylinder, respectively; and *A* is the piston area.

**Figure 6:**
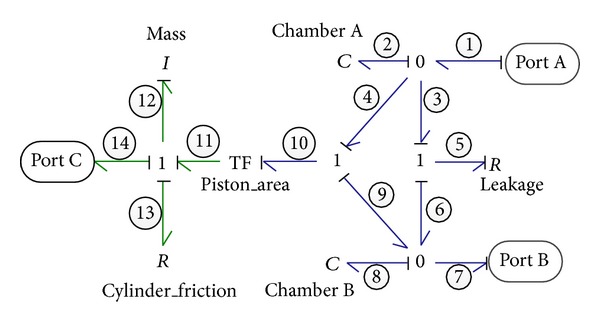
Bond graph model of the cylinder.

In this particular system, the two hydraulic cylinders are the energy regenerative components, recovering and converting the hydraulic energy of emulsion fluid output from emulsion pump first into mechanical energy and then back into hydraulic energy of oil. A couple of symmetrical cylinders are employed to make sure that the output force and velocity of cylinder remain the same no matter which chamber of the cylinder is filled with pressurized fluid. As illustrated in Figure 6, a sophisticated model of the symmetrical cylinder is established. This model contains three interact ports with the environment: port A and port B are the inflow and outflow ports of hydraulic domain, while port C is the velocity output port of mechanical domain. Losses associated with the internal leakage and friction at the piston are implemented using the resistive elements *R*_5_ and *R*_13_, respectively. The two capacitive elements *C*_2_ and *C*_8_ are used to represent the compressibility of fluid in the two chambers of cylinders, the capacitance of which keeps changing during operation due to the change of chamber volume caused by the piston movement.

The state-space equations of cylinder derived from its bond graph could be expressed as

(11) Q˙2=−1C2R5Q2+1C8R5Q8−ApiI12P12+f1,Q˙8=1C2R5Q2−1C8R5Q8+ApiI12P12−f7,P˙12=−ApiC2Q2+ApiC8Q8−R13I12P12+e14.

In the above, *Q*_2_, ${\dot{Q}}_{2}$ and *Q*_8_, ${\dot{Q}}_{8}$ are the volume and flow rate of the two chambers of cylinder, respectively; *P*_12_ and ${\dot{P}}_{12}$ are the output momentum and force of cylinder, respectively; and *f*_1_, *f*_7_, and *e*_14_ are the input flow rate, output flow rate, and output force of cylinder, respectively.

#### 2.2.6. Rectifier Valve

The rectifier valve, which resembles a full-wave rectifier circuit in electrical system, consists of four check valves connected in a certain pattern. As known, the check valve allows flow in only one direction, like a diode in electrical systems. Such behavior can be modeled in bond graph using a MR element with conditional equations which determine the flow through the valve depending on the pressure difference between the two ports. The equation of one check valve is given as follows:

(12) $$\begin{array}{c} q_{c}=\{\begin{array}{cc} \frac{\Delta p}{p_{\mathrm{cl}}}q_{\mathrm{cl}} & \text{if}\;\;\Delta p<p_{\mathrm{cl}}\\ q_{\mathrm{cl}}+\frac{\Delta p-p_{\mathrm{cl}}}{p_{\text{op}}-p_{\mathrm{cl}}}\left(q_{\text{op}}-q_{\mathrm{cl}}\right) & \text{if}\;\;p_{\mathrm{cl}}\leq \Delta p\leq p_{\text{op}}\\ C_{d}A_{c}\sqrt{\frac{2\Delta p}{\rho }} & \text{if}\;\;\Delta p>p_{\text{op}}, \end{array} \end{array}$$

where *q*_*c*_ and Δ*p* are the flow-rate and pressure difference of check valve, respectively; *p*_cl⁡_ and *p*_op_ are reference pressures for the closing and opening of the valve; *q*_cl⁡_ and *q*_op_ are the corresponding flow rate; *C*_*d*_ is the discharge coefficient of valve; *A*_*c*_ is the cross-section area of valve; and *ρ* is the fluid density.

**Figure 7:**
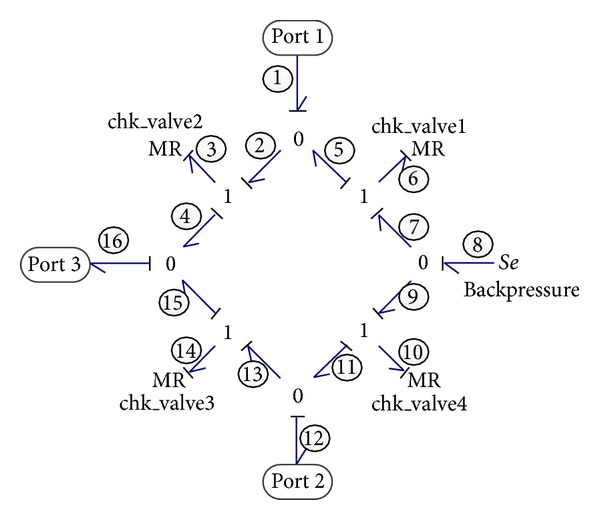
Bond graph model of the rectifier valve.

As shown in Figure 7, the bond graph model of the rectifier valve contains three ports: port 1 and port 2 are linked to the two chambers of the recovery cylinder, whereas port 3 is connected to VDHM, transferring the recovered energy back into the system again.

The flow rate of the rectifier valve could be expressed as

(13) f1=(1MR3+1MR6)e1−1MR6e8−1MR3e16,f8=−1MR6e1+(1MR10+1MR6)e8−1MR10e12,f12=1MR10e8−(1MR10+1MR14)e12+1MR14e16,f16=1MR3e1+1MR14e12−(1MR3+1MR14)e16,

where *f*_1_, *e*_1_, *f*_12_, and *e*_12_ are the flow rate and pressure of port 1 and port 2, respectively; *f*_8_ and *e*_8_ are flow rate and pressure of oil supply, respectively; and *f*_16_ and *e*_16_ are the flow rate and pressure of port 3, respectively.

#### 2.2.7. Accumulator

The accumulator, like a capacitor in electrical systems, could smooth the pressure and flow fluctuations in the system as well as store hydraulic energy. The accumulator employed in this system is a common gas-charged one which uses a gas-filled bladder in its chamber. In practical applications, the gas chamber of the accumulator is precharged to a certain pressure before installation and therefore the accumulator only functions when the fluid pressure surpasses the gas pressure. When the accumulator is being charged (or discharged), the compression (or expansion) of the gas follows the following relation:

(14) $$\begin{array}{c} p_{2}=p_{1}{\left(\frac{V_{1}}{V_{1}-\Delta V}\right)}^{k}, \end{array}$$

where *p*_1_ and *V*_1_ are the initial gas pressure and volume, respectively; Δ*V* is the fluid volume that flowed into the accumulator; and *p*_2_ is the charged pressure of accumulator. The value of the specific heat ratio *k* depends on whether the expansion and compression occur rapidly or slowly.

**Figure 8:**
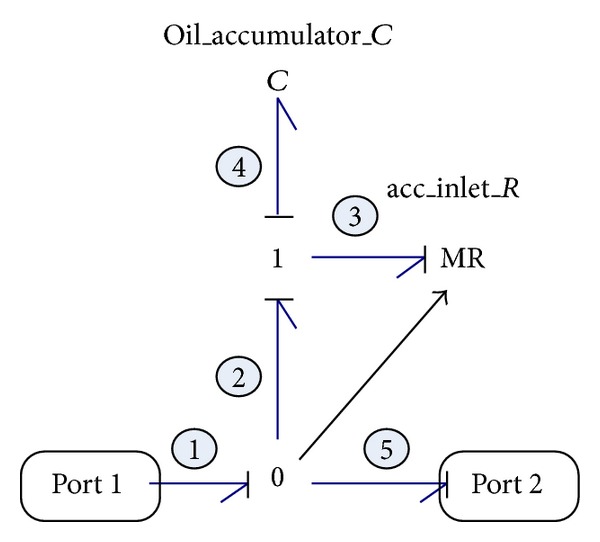
Bond graph model of the accumulator.

The bond graph model of an accumulator is illustrated in Figure 8. The MR element used here not only represents the flow resistance at the inlet port of accumulator when the accumulator interacts with the whole system, being charged or discharged, but also functions like a switch which could isolate the accumulator from the system if the fluid pressure does not surpass the precharged gas pressure. The resistance of MR is modulated by the control signal of inlet pressure symbolized by a triangle arrow in Figure 8.

The state-space equation of accumulator derived from Figure 8 could be expressed as follows:

(15) $$\begin{array}{c} {\dot{Q}}_{4}=-\frac{1}{C_{4}MR_{3}}Q_{4}+\frac{1}{MR_{3}}e_{1}, \end{array}$$

where *Q*_4_ and ${\dot{Q}}_{4}$ are the volume and flow rate of fluid in the accumulator, respectively, and *e*_1_ is the input fluid pressure of accumulator.

#### 2.2.8. Pipelines

**Figure 9:**
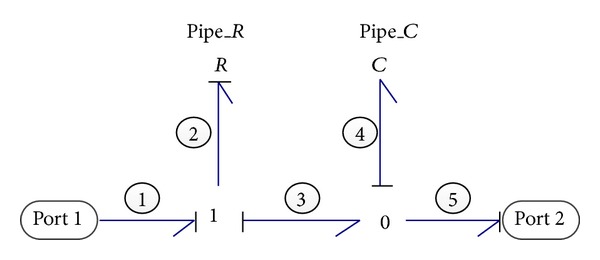
Bond graph model of the pipeline.

Pipelines, similar to transmission lines in electrical systems, transfer hydraulic fluid from one point to another. The bond graph model of pipelines is illustrated in Figure 9. There are two significant characteristics of pipes which should be considered in modeling this system. One is the energy loss which happens by means of pressure drops as the fluid flows through the pipelines. This pressure drop along the pipes can be symbolized by a resistive element and expressed as

(16) $$\begin{array}{c} R=\frac{128\rho \upsilon L}{\pi D^{4}}, \end{array}$$

where *ρ* is the fluid density; *υ* is the kinematic viscosity of the fluid; *L* is the pipe length; and *D* is the pipe diameter.

The other one is the capacitance effect of the fluid inside the pipes represented by a *C* element, like the chamber of a cylinder. However, the inertia effect of fluid in pipelines is reasonably neglected because flow-rate fluctuations of high frequency do not exist in this system during operation.

The state-space equation of the pipeline is derived as follows:

(17) $$\begin{array}{c} {\dot{Q}}_{4}=-\frac{1}{C_{4}R_{2}}Q_{4}+\frac{1}{R_{2}}e_{1}-f_{5}, \end{array}$$

where *Q*_4_ and ${\dot{Q}}_{4}$ are the volume and flow-rate of fluid inside the pipelines, respectively, and *e*_1_ and *f*_5_ are the input pressure and output flow rate of the pipelines, respectively.

### 2.3. System Modeling

**Figure 10:**
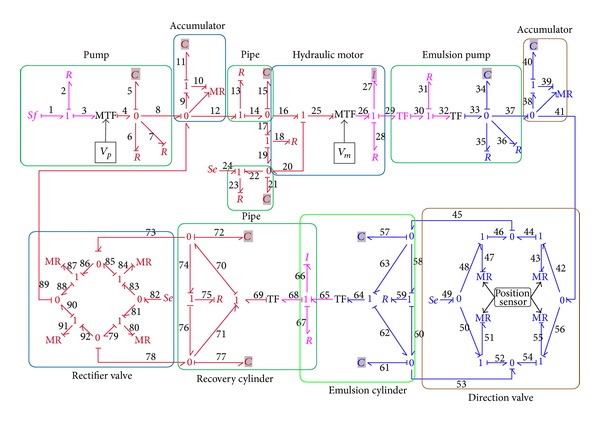
Bond graph model of the overall system.

The bond graph model of the overall system, as shown in Figure 10, is established by assembling all the components models according to the hydraulic system in Figure 1. In Figure 10, the bonds and elements in magenta represent the power transferring and distributions in mechanical domain, while the bonds and elements in red and blue symbolize the power transferring and distributions in hydraulic domain of oil and emulsion fluid, respectively.

Some simplifications and approximations are made in the overall model to avoid the causality conflicts of energy store elements (*C* and *I*) and hence the differential algebraic equations. Further information about causal conflicts and solutions could be referred to in [32, 33]. The *I*_66_ element, for instance, represents the combined mass of pistons and rods of both cylinders. Similarly, the *I*_27_ element represents the combined moment of inertia of both hydraulic motor and emulsion pump. The overall bond graph model of this system contains 12 energy store elements (*C* and *I* elements) and hence the state-space equations derived from this model consist of 12 state variables and 12 equations. The state-space equation of the overall system in matrix can be expressed as

(18) $$\begin{array}{c} {\left[\dot{x}\left(t\right)\right]}_{12\times 1}={\left[A\right]}_{12\times 12}•{\left[x\left(t\right)\right]}_{12\times 1}+{\left[B\right]}_{12\times 4}•{\left[u\left(t\right)\right]}_{4\times 1}, \end{array}$$

where [**x**(*t*)]_12×1_ = [*Q*_5_ *Q*_11_ *Q*_15_ *Q*_21_ *P*_27_ *Q*_34_ *Q*_40_ *Q*_57_ *Q*_61_ *P*_66_ *Q*_72_ *Q*_77_]^*T*^; ${\left[\dot{x}(t)\right]}_{12\times 1}$ is the time differential of [**x**(*t*)]_12×1_; and [**u**(*t*)]_4×1_ = [*f*_1_ *e*_24_ *e*_49_ *e*_82_]^*T*^. The detailed [**A**]_12×12_ and [**B**]_12×4_ are presented in Appendix.

## 3. Simulation of ERS

The simulation of the bond graph model of the overall system was carried out within the MATLAB/Simulink software. The durable test in the test standard, for instance, was chosen for simulation which required the test emulsion pump to be running continuously and constantly at its nominal pressure and speed for 500 hours. The nominal pressure and speed of the test emulsion pump used in simulation are 20 MPa and 1470 rev/min, and the major parameters of simulation are shown in Table 1.

**Figure 11:**
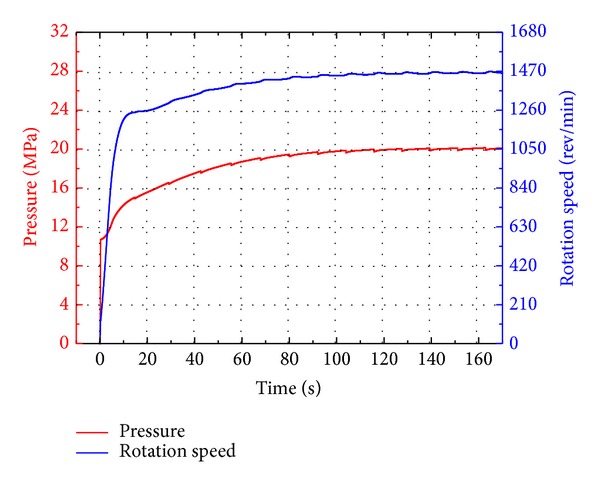
Simulation results of the test emulsion pump.

**Figure 12:**
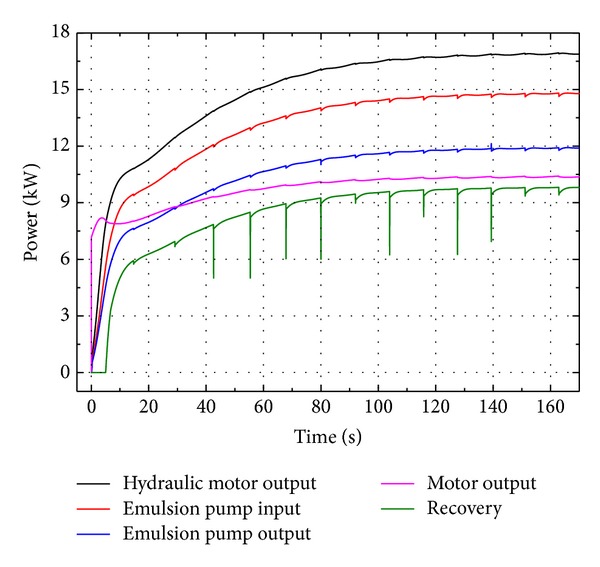
Power consumption of major components by simulation.

**Figure 13:**
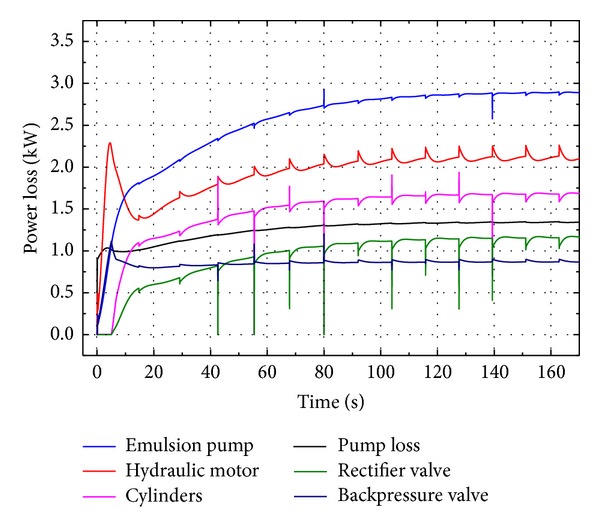
Power loss comparison of major components by simulation.

During the simulation the geometric displacements of both VDP and VDHM were kept constant to ensure that the emulsion pump will be running at the nominal condition as the test standard required. The simulation was carried out for 160 s with a maximum step of 0.15 s, simulating the ERS starting from static stage to final steady dynamic stage. The simulation results are depicted in Figures 11–13.

As illustrated in Figure 11, it takes nearly 80 s for the ERS to establish its final steady state due to the two accumulators of which the precharged gas pressures are much lower than the steady ones. In the steady stage, the rotation speed is around 1470 rev/min and the pressure is 20 MPa, satisfying the requirements of durable test.

More importantly the power consumptions of major components should be investigated in depth, as depicted in Figure 12. On one hand, it is the output power of emulsion pump that can be recovered and reutilized from the durable test by this ERS but wasted by the original system in the test standard. This total recoverable power is around 11.93 kW, while the regenerative power saved by this ERS is about 9.74 kW. Therefore, the regenerative coefficient of this system (the ratio of the recovered power to output power of emulsion pump) reaches as high as 81.6%. The remaining 18.4% of 11.93 kW is cost by means of frictions and pressure losses in cylinders and rectifier valve.

On the other hand, the overall energy efficiency of the whole system is also of great significance for ERS. From the global energy point of view, the actual power consumption of this ERS for durable test, that is, the output power of induction motor, is about 10.36 kW, whereas the input power of emulsion pump, that is, the power cost and wasted by the original test system, is 14.83 kW. In other words, when doing durable tests, this novel ERS costs only 10.36 kW instead of the original 14.83 kW, saving 4.47 kW. Consequently the energy-saving coefficient of this system (the ratio of decreased power consumption to original power consumption) is as high as 30.1%.

Figure 13 illustrates the comparison of power losses between major components. As seen in Figure 13, emulsion pump costs the most power loss (2.90 kW) due to its volumetric and mechanical coefficient and accounts for 28.0% of the total power loss (10.36 kW). The power losses of VDHM and VDP are 2.12 kW and 1.34 kW and account for 20.5% and 12.9% of the total loss, respectively. The two cylinders cost 1.67 kW (16.1%), while the rectifier valve costs 1.15 kW (11.1%). The power loss of backpressure valve is 0.88 kW and accounts for 8.5% of the total power loss.

These losses caused by the internal friction and leakage of hydraulic components, however, could be reduced by employing hydraulic components with higher machining accuracy quality and hence higher efficiency, achieving a better energy efficiency of the overall system and saving more energy.

## 4. Experimental Validation

**Figure 14:**
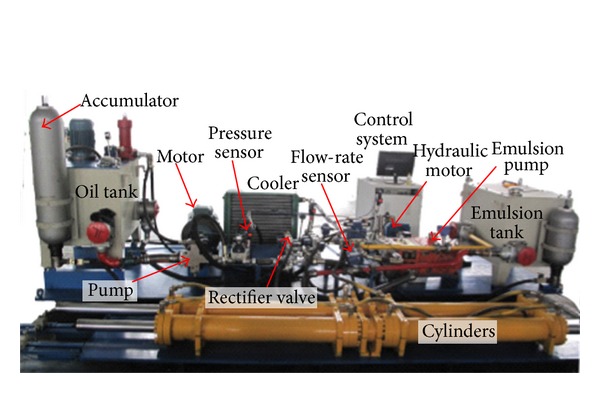
Test bench.

**Figure 15:**
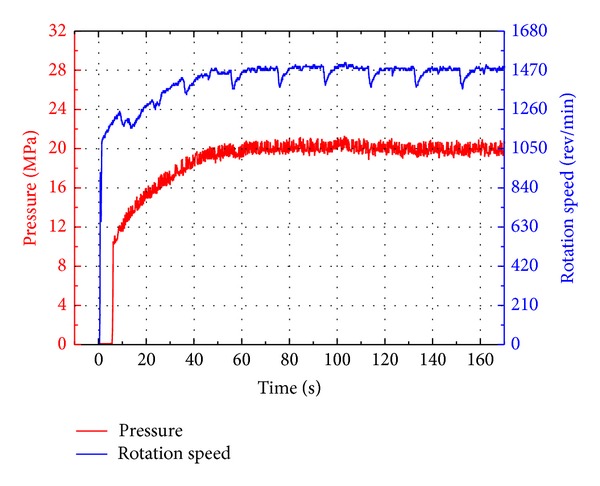
Experiment results of the test emulsion pump.

**Figure 16:**
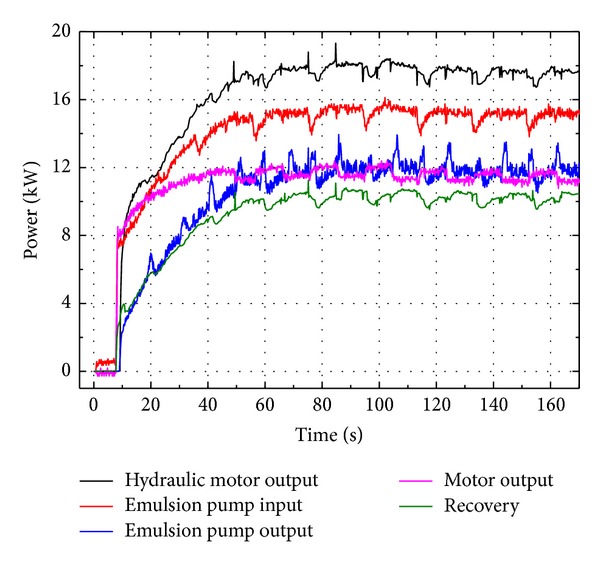
Power consumption of major components by experiment.

**Figure 17:**
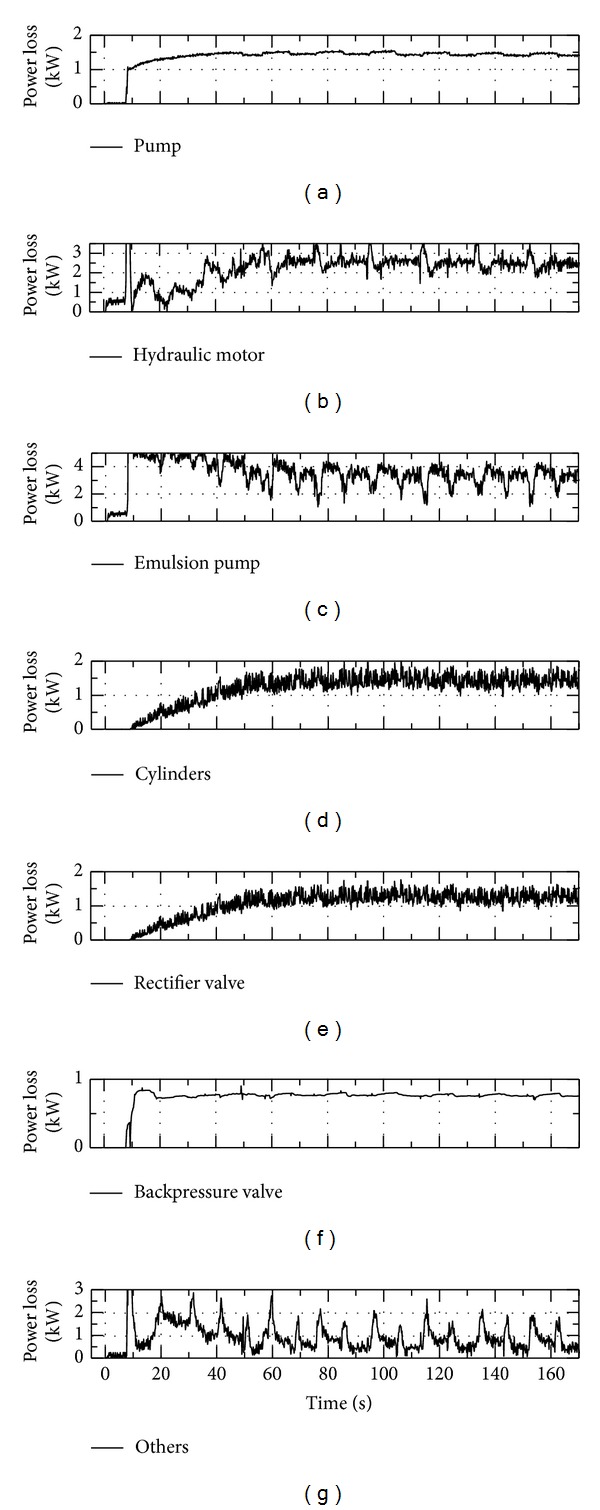
Power loss comparison of major components by experiment.

Experiments were carried out to validate the bond graph modeling and simulation results of the novel ERS. This test bench, as shown in Figure 14, was built according to the schematic diagram in Figure 1. For validation conveniences, major parameters of the test bench are the same as those of the simulation shown in Table 1. Like the simulation, the whole system was driven by the electric motor from the state of rest, and during experimental tests the geometric displacements of both VDP and VDHM were kept constant to ensure that the emulsion pump will be running at the nominal condition as the test standard required. The experiment was also carried out for 160 s with a sample time of 0.15 s. The experiment results are depicted in Figures 15–17.

In Figure 15, the speed and pressure curves illustrate that the test emulsion pump is running at its nominal speed (1470 r/min) and pressure (20 MPa), satisfying the durable test requirements and verifying that both the bond graph modeling and simulation of this novel ERS are accurate.

Due to the buffering of two accumulators, the system gradually establishes its final steady state in around 65 seconds. The periodic fluctuations in speed curves are caused by the motion direction changes of the cylinders. When cylinders come to the stroke end, position sensors trigger the direction control valve to change from one working position to the other one, changing the flow paths. These transient flow paths changes result in the flow-rate loss of cylinders and rectifier valve, and hence the input flow-rate of VDHM decreases, so does the output speed of VDHM (also the speed of emulsion pump). After comparison and analysis, we find that these periodic fluctuations could be better buffered by accumulators of larger volume.

The experiment results of power consumptions are depicted in Figure 16. The output power of emulsion pump is around 11.80 kW, while the regenerative power saved by this ERS is about 10.18 kW. Therefore, the experimental regenerative coefficient of this system (the ratio of the recovered power to output power of emulsion pump) is as high as 86.3%. The output power of induction motor is 11.43 kW, whereas the input power of emulsion pump is 15.02 kW. So the power saved by experimental tests is 3.59 kW and the energy-saving coefficient by experiment is 23.9%.

Figure 17 illustrates the detailed power losses of major components. As shown in Figure 17, emulsion pump costs the most power loss (3.22 kW) due to its efficiency and accounts for 28.2% of the total power loss (11.43 kW). The average power losses of VDHM and VDP are 2.51 kW and 1.44 kW and account for 22.0% and 12.6% of the total loss, respectively. The two cylinders cost 1.41 kW (12.3%), while the rectifier valve costs 1.24 kW (10.8%). The power loss of backpressure valve is 0.77 kW and accounts for 6.7% of the total power loss.

These experimental results in both power consumption and loss reasonably match those of simulation with little errors and consequently validate the accuracy of bond graph modeling and simulation of the novel system.

## 5. Concluding Remarks

This paper demonstrates the virtue of bond graph methodology in modeling the energy regenerative system for emulsion pump tests, a system involving several energy domains and energy conversion processes, by first considering the components separately and then assembling them together accordingly. Bond graph models of each component are developed and the state-space equations of each component are also derived, so are the bond graph model and the state-space equations of the whole system.

Illustrative simulation results of the novel ERS for the durable test have been represented. The simulation results of the model validate that this ERS not only fulfills the requirements of the test but also recovers and reutilizes 81.6% of the output energy of the test emulsion pump which is wasted by the original test system. Moreover, this novel ERS could reduce 30.1% energy consumption as compared to the original test system. Experiments are also carried out and the results primely validate the accuracy of both the bond graph modeling and simulation of this novel ERS.

Although it has periodic fluctuations of speed and its energy efficiency is not perfectly high enough, this ERS, we believe, does provide a promising method of energy saving for emulsion pump tests. In future, we would like to use the described bond graph model to develop online control algorithms for this ERS.

## Figures and Tables

**Table 1:** Simulation parameters.

Parameter	Value	Unit
Motor rotation speed	1470	rev/min
Pump displacement	23.9	mL/rev
Hydraulic motor displacement	42.0	mL/rev
Mechanical efficiency of pump and hydraulic motor	0.95	—
Volumetric efficiency of pump and hydraulic motor	0.91	—
Emulsion pump displacement	73.1	mL/rev
Nominal input rotation speed of emulsion pump	1470	rev/min
Internal reduction ratio	1470/547	—
Crankshaft speed of emulsion pump	547	rev/min
Mechanical efficiency of emulsion pump	0.9	—
Volumetric efficiency of emulsion pump	0.9	—
Cylinder piston area	10725	mm^2^
Cylinder stroke	0.75	m
Back pressure	1.4	MPa
Precharged gas pressure of accumulator	10.5	MPa
Accumulator volume	10	L

## Appendix

The nonzero item of [**A**]_12×12_ and [**B**]_12×4_ is as follows:

(A.1) $$\begin{array}{c} a_{1,1}=-\frac{1}{C_{5}}\left(\frac{1}{R_{6}}+\frac{1}{R_{7}}+\frac{1}{MR_{10}}+\frac{1}{R_{13}}+\frac{1}{MR_{87}}+\frac{1}{MR_{91}}\right),\\ a_{1,2}=\frac{1}{MR_{10}C_{11}},\;\;a_{1,3}=\frac{1}{R_{13}C_{15}},\\ a_{1,11}=\frac{1}{C_{72}MR_{87}},\;\;a_{1,12}=\frac{1}{C_{77}MR_{91}};\\ a_{2,1}=\frac{1}{C_{5}MR_{10}},\;\;a_{2,2}=-\frac{1}{MR_{10}C_{11}};\\ a_{3,1}=\frac{1}{C_{5}R_{13}},\;\;a_{3,3}=-\frac{1}{C_{15}}\left(\frac{1}{R_{13}}+\frac{1}{R_{18}}\right),\\ a_{3,4}=\frac{1}{R_{18}C_{21}},\;\;a_{3,5}=-\frac{V_{m}}{I_{27}};\\ a_{4,3}=-\frac{1}{C_{15}R_{18}},\;\;a_{4,4}=-\left(\frac{1}{R_{18}}+\frac{1}{R_{23}}\right),\\ a_{4,5}=-\frac{V_{m}}{I_{27}};\\ a_{5,3}=\frac{V_{m}}{C_{15}},\;\;a_{5,4}=-\frac{V_{m}}{C_{21}},\\ a_{5,5}=\left(-R_{28}+iR_{31}\right)\frac{1}{I_{27}},\;\;a_{5,6}=-\frac{iV_{\text{ep}}}{C_{34}};\\ a_{6,5}=\frac{iV_{\text{ep}}}{I_{27}},\\ a_{6,6}=\frac{1}{C_{34}}\left(-\frac{1}{R_{35}}-\frac{1}{R_{36}}-\frac{1}{R_{39}}+\frac{1}{MR_{43}}+\frac{1}{MR_{55}}\right),\\ a_{6,7}=\frac{1}{R_{39}C_{40}},\;\;a_{6,8}=-\frac{1}{MR_{43}C_{57}},\\ a_{6,9}=-\frac{1}{MR_{55}C_{61}};\\ a_{7,6}=\frac{1}{C_{34}R_{39}},\;\;a_{7,7}=-\frac{1}{R_{39}C_{40}};\\ a_{8,6}=\frac{1}{C_{34}MR_{43}},\\ a_{8,8}=\frac{1}{C_{57}}\left(-\frac{1}{MR_{43}}+\frac{1}{MR_{47}}-\frac{1}{R_{59}}\right),\\ a_{8,9}=\frac{1}{R_{59}C_{61}},\;\;a_{8,10}=\frac{A}{I_{66}};\\ a_{9,6}=-\frac{1}{C_{34}MR_{55}},\;\;a_{9,8}=\frac{1}{C_{57}R_{59}},\\ a_{9,9}=\frac{1}{C_{61}}\left(-\frac{1}{MR_{51}}+\frac{1}{MR_{55}}+\frac{1}{R_{59}}\right),\\ a_{9,10}=\frac{A}{I_{66}};\\ a_{10,8}=\frac{A}{C_{57}},\;\;a_{10,9}=-\frac{A}{C_{61}},\\ a_{10,10}=-\frac{R_{67}}{I_{66}},\;\;a_{10,11}=-\frac{A}{C_{72}},\\ a_{10,12}=\frac{A}{C_{77}};\\ a_{11,1}=\frac{1}{C_{5}MR_{87}},\;\;a_{11,10}=\frac{A}{I_{66}},\\ a_{11,11}=-\frac{1}{C_{72}}\left(\frac{1}{R_{75}}+\frac{1}{MR_{84}}+\frac{1}{MR_{87}}\right),\\ a_{11,12}=\frac{1}{R_{75}C_{77}};\\ a_{12,1}=\frac{1}{C_{5}MR_{91}},\;\;a_{12,10}=-\frac{A}{I_{66}},\\ a_{12,11}=\frac{1}{C_{72}R_{75}},\\ a_{12,12}=-\frac{1}{C_{77}}\left(\frac{1}{R_{75}}+\frac{1}{MR_{80}}+\frac{1}{MR_{91}}\right);\\ b_{1,1}=V_{p},\;\;b_{4,2}=\frac{1}{R_{23}},\\ b_{8,3}=-\frac{1}{MR_{47}},\;\;b_{9,3}=\frac{1}{MR_{51}},\\ b_{11,4}=\frac{1}{MR_{84}},\;\;b_{12,4}=\frac{1}{MR_{80}}. \end{array}$$
